# Mode of Birth and Stroke Risk After Childbirth Among Women With Moyamoya Disease

**DOI:** 10.1001/jamanetworkopen.2026.3112

**Published:** 2026-03-23

**Authors:** Jong Hun Kim, Kwan Heup Song, Man Young Park, Sang Hun Lee, Jae-woo Lee, Ho Yeon Kim, Jin-Man Jung

**Affiliations:** 1 Department of Neurology, Korea University Ansan Hospital, Ansan, Republic of Korea; 2 Department of Obstetrics and Gynecology, Korea University Ansan Hospital, Ansan, Republic of Korea; 3 Division of Digital Clinical Research, Korea Institute of Oriental Medicine, Daejeon, Republic of Korea; 4 Department of Family Medicine, Chungbuk National University Hospital, Cheongju, Republic of Korea; 5 Department of Family Medicine, Chungbuk National University College of Medicine, Cheongju, Republic of Korea; 6 Korea University Zebrafish Translational Medical Research Center, Ansan, South Korea

## Abstract

**Importance:**

There is limited large-scale evidence to guide the optimal mode of birth for patients with moyamoya disease (MMD).

**Objective:**

To evaluate whether the mode of birth (cesarean vs vaginal) is associated with stroke risk after childbirth for women with MMD.

**Design, Setting, and Participants:**

This cohort study evaluated stroke outcomes up to 3 years after childbirth. A nationwide, population-based analysis was performed using data from the Health Insurance Review and Assessment Service of South Korea. Individuals with MMD from January 1, 2002, to December 31, 2023 were identified. Among 31 750 patients, those with birth-related procedure codes were selected. The study population was restricted to women aged 19 to 49 years, and those with a diagnosis of malignant neoplasm within 3 years before the index date (date of childbirth) were excluded. Data were analyzed from June 11 to September 8, 2025.

**Exposure:**

Mode of birth.

**Main Outcomes and Measures:**

The primary outcome was any stroke, defined as a composite of ischemic or hemorrhagic stroke. Secondary outcomes included ischemic stroke, hemorrhagic stroke, and transient ischemic attack.

**Results:**

Of 1683 women analyzed (mean [SD] age, 33.6 [7.8] years), 1077 (64.0%) had cesarean births, and 606 (36.0%) had vaginal births. Post partum (3 months), any stroke incidence was 63.49 and 33.33 per 1000 person-years for cesarean and vaginal births, respectively. Multivariable analyses showed no significant risk differences for any stroke by birth mode at 3 months (adjusted hazard ratio [aHR], 0.71 [95% CI, 0.26-1.97]; *P* = .52) or 3 years (aHR, 0.90 [95% CI, 0.55-1.47]; *P* = .67). A significant interaction was observed between the mode of birth and the clinical onset type of MMD for the risk of any stroke (interaction *P* = .04 after Bonferroni correction); the adjusted HR for vaginal vs cesarean birth was 0.10 (95% CI, 0.01-0.79) in the asymptomatic or nonvascular onset subgroup, 1.49 (95% CI, 0.73-3.03) in the ischemic onset subgroup, and 0.94 (95% CI, 0.50-1.77) in the hemorrhagic onset subgroup. Notably, stroke incidence peaked in the early postpartum period (≤6 months: 35.7 per 1000 person-years), decreased at 1 year, and thereafter remained at a similar level.

**Conclusions and Relevance:**

In this cohort study of women with MMD, MMD itself was not found to be an absolute indication for cesarean birth; birth planning should be individualized based on obstetric factors and clinical onset type rather than routine preference for cesarean birth. In addition, vigilant monitoring and preventive strategies during the early postpartum period are warranted.

## Key Points

**Question:**

Is the mode of birth (cesarean vs vaginal) associated with differences in the risk of postpartum stroke among women with moyamoya disease?

**Findings:**

In this cohort study of 1683 women with moyamoya disease, the overall risk of postpartum stroke did not significantly differ by mode of birth. However, the effect of the mode of birth was significantly modified by the patient’s clinical onset type (eg, hemorrhagic, ischemic, or asymptomatic or nonvascular).

**Meaning:**

These findings suggest that a routine preference for cesarean birth among women with moyamoya disease may be unnecessary.

## Teaser

This cohort study evaluates whether the mode of birth (cesarean vs vaginal) is associated with stroke risk after childbirth among women with moyamoya disease using data from the Health Insurance Review and Assessment Service of South Korea from 2002 to 2023.

## Introduction

Moyamoya disease (MMD) is a progressive cerebrovascular disorder characterized by stenosis and occlusion of the terminal internal carotid arteries, leading to reduced cerebral blood flow and prompting fragile collateral (“moyamoya”) vessel formation.^1,2,3,4,5^ Although rare globally, MMD is increasingly recognized worldwide^6^ and more prevalent in East Asia (incidence, 6.0-16.1 per 100 000 in Korea and Japan).^7,8^ Its rarity hinders research, limiting understanding and guidelines for clinical decisions.^6^ MMD primarily affects women of childbearing age—1.8-fold predominance among women and 90% cases presenting before 40 years of age.^7,9^ A substantial proportion of patients may encounter the profound hemodynamic changes associated with pregnancy and childbirth, making peripartum management a critical clinical concern in MMD care. Nevertheless, evidence on MMD pregnancy and childbirth management is largely limited to small case reports or single-center studies,^10,11,12,13,14,15,16^ underscoring the need for large-scale research to inform clinical care.

One of the most significant unresolved issues in MMD management is determining the optimal mode of birth. During labor, factors such as maternal pain, Valsalva maneuver, and hyperventilation can adversely affect cerebral hemodynamics, leading to cesarean birth as the preferred method for women with MMD to date.^17,18,19^ In addition, hypertensive disorders may occur more frequently among women with MMD.^20^ However, studies indicate that during childbirth and the postpartum period, most cerebrovascular complications in women with MMD are ischemic strokes rather than hemorrhagic strokes.^19^ Cesarean birth can exacerbate hypercoagulability and induce hypovolemia, thereby increasing the risk of an ischemic event.^21,22,23^ Some studies therefore suggest that vaginal birth may be preferable when accompanied by strict monitoring, adequate pain control, and assisted vaginal birth.^10,11,12,13,15,16,18^

This nationwide, population-based cohort study examines childbirth among women with MMD, presenting detailed incidence rates across peripartum and long-term follow-up periods. To date, giving birth is not considered a contraindication for women with MMD.^19,24,25,26^ However, quantitative evidence on stroke risk by mode of birth remains limited.^17,27,28^ Herein, we compare the incidences of ischemic stroke, hemorrhagic stroke, and transient ischemic attack (TIA) between women with cesarean birth and those with vaginal birth to provide precise and objective evidence. Our aim is to provide clinically actionable data to guide the mode-of-birth decisions of women with MMD.

## Methods

### Study Design and Setting

This nationwide retrospective cohort study used claims data from the Health Insurance Review and Assessment Service (HIRA) in South Korea, covering the period from January 1, 2002, to December 31, 2023. The HIRA database includes health care services reimbursed by the National Health Insurance Service and contains comprehensive medical information, including diagnostic codes, procedure and surgery codes, prescription records, demographic data, and health care professional types. HIRA covers nearly the entire South Korean population, rendering this dataset highly nationally representative. Data were provided by HIRA, and the study was conducted in accordance with the ethical principles of the Declaration of Helsinki. This study followed the Strengthening the Reporting of Observational Studies in Epidemiology (STROBE) reporting guideline for cohort studies. This study was approved by the institutional review board of Korea University Ansan Hospital. Because only deidentified secondary data were used, the requirement for informed consent was waived.

### Study Population

MMD was defined by an *International Statistical Classification of Diseases and Related Health Problems, Tenth Revision* (*ICD-10*) diagnosis code I67.5 recorded as a diagnosis, along with a Special Calculation System for Patients With Severe Diseases code V128. Patients with a diagnosis of MMD were initially identified. Women with pregnancy-related diagnostic codes were selected. The cohort was then restricted to women aged 19 to 49 years who had birth records. To minimize confounding, patients with a history of malignant neoplasms (*ICD-10* codes C00-C97) within 3 years before the index date (date of childbirth) were excluded. Women with MMD who gave birth were included in the final analysis. Only first births were considered, and cases of subsequent births were excluded. The patient selection process and stepwise exclusions are illustrated in the Figure.

**Figure.**
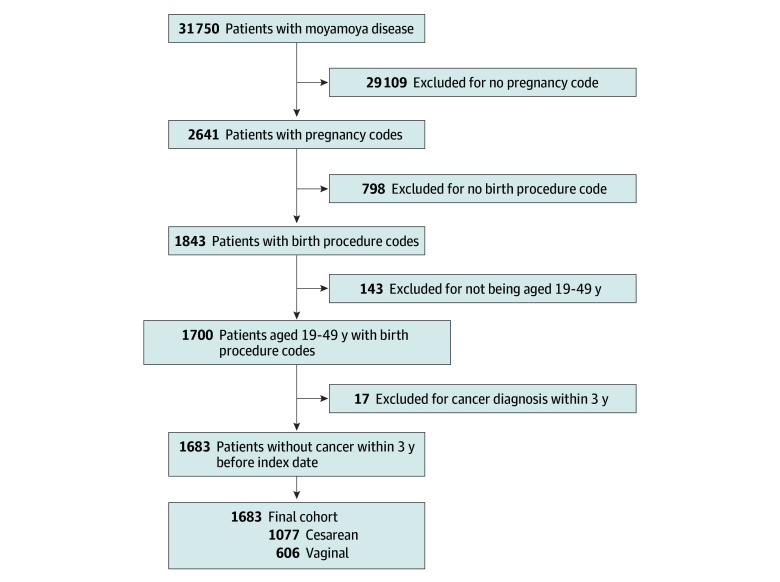
Flowchart of Study Population Selection

### Variables and Outcome Definitions

The primary exposure was the mode of birth, classified as vaginal (codes O75.7, O80, O81, O83, O84, and O84.1) or cesarean (codes O82 and O84.2) based on procedure codes, with date of childbirth defined as the index date. The primary outcome was incidence of any stroke (ischemic or hemorrhagic) within 3 years after giving birth. Secondary outcomes included incidences of ischemic stroke (*ICD-10* code I63), hemorrhagic stroke (codes I60-I62), and TIA (code G45) during the same follow-up period. Pregnancy-related complications (eg, multiple pregnancy, preeclampsia, hypertensive disorders, gestational or overt diabetes, and eclampsia) were identified using *ICD-10* codes. All stroke and TIA outcomes required a corresponding hospitalization record with evidence of brain imaging performed within ±7 days of admission. MMD onset was categorized as ischemic, hemorrhagic, or asymptomatic or nonvascular (eg, headache or seizure) at diagnosis, and timing of diagnosis was classified as prepregnancy, during pregnancy, puerperium (≤6 weeks post partum), or postpuerperium (>6 weeks post partum). Data on bypass surgery type (direct, indirect, or combined) and medication use within 120 days before giving birth were also collected. The full list of *ICD-10* codes and definition of the variables were provided in eTable 1 in Supplement 1.

### Statistical Analysis

Baseline characteristics were summarized as mean (SD) values or frequencies (percentages) and compared using the *t* test or the χ^2^ test. Cox proportional hazards models were used to estimate associations between the mode of birth and stroke outcomes, with the proportional hazards assumption tested using Schoenfeld residuals. Follow-up time was measured from date of childbirth. Risks were compared across prespecified intervals (0-3, 0-12, and 0-36 months). Three models were constructed: model 1 included the mode of birth only; model 2 was additionally adjusted for maternal age; and model 3 was further adjusted for MMD diagnostic timing and onset type, pregnancy-related complications, prior stroke, comorbidities, bypass surgery, and medication use. Covariates are described in eTable 1 in Supplement 1. Cumulative risks were estimated for each interval, and piecewise Cox regression was used to evaluate time-varying effects (≤180, 181-365, 366-730, and >730 days).^29^ Prespecified subgroup analyses included maternal age, timing and onset type of MMD, bypass surgery, and revascularization type. A 2-sided *P* < .05 was considered statistically significant for the primary outcome (any stroke). For the 3 secondary outcomes and 5 subgroup analyses, the Bonferroni correction was applied, setting significance thresholds at *P* < .02 and *P* < .01, respectively. Analyses for subarachnoid and intracerebral hemorrhage were exploratory; thus, no additional multiplicity correction was applied, but the secondary outcome threshold (*P* < .02) was maintained. Because the nationwide claims database provided complete data, no imputation was required. Analyses were performed using R, version 3.5.1 (R Foundation for Statistical Computing). Data were analyzed from June 11 to September 8, 2025.

## Results

### Baseline Characteristics of the Study Population

A total of 31 750 patients with a diagnosis of MMD were initially identified. Among these, 2641 women with pregnancy-related diagnostic codes were selected. The cohort was then restricted to women aged 19 to 49 years who had birth records, yielding 1700 participants. To minimize confounding, 17 patients with a history of malignant neoplasms (*ICD-10* codes C00-C97) within 3 years before the index date (date of childbirth) were excluded. A total of 1683 women with MMD were included in the analysis (mean [SD] age, 33.6 [7.8] years), of whom 1077 (64.0%) underwent cesarean birth and 606 (36.0%) underwent vaginal birth (Table 1). The mean (SD) age was lower in the cesarean group than in the vaginal group (32.2 [8.0] years vs 36.1 [6.6] years; *P* < .001), with a smaller proportion of younger women (19-29 years: 318 [29.5%] vs 230 [38.0%]) and a larger proportion of older women (40-49 years: 51 [4.7%] vs 11 [1.8%]). Distributions of MMD onset type were similar between groups (asymptomatic or nonvascular: 685 [63.6%] vs 397 [65.5%]; hemorrhagic: 130 [12.1%] vs 74 [12.2%]; ischemic: 262 [24.3%] vs 135 [22.3%]; *P* = .63), but diagnosis timing differed (prepregnancy: 489 [45.4%] vs 52 [8.6%]; postpuerperium: 508 [47.2%] vs 537 [88.6%]; *P* < .001). Rates of bypass surgery were similar between groups (301 [27.9%] vs 152 [25.1%]; *P* = .22), although the distribution of bypass surgery types differed significantly (*P* = .02). The cesarean group had higher rates of diabetes (16 [1.6%] vs 1 [0.2%]; *P* = .02), heart failure (34 [3.3%] vs 7 [1.2%]; *P* = .01), hypertension (25 [2.5%] vs 4 [0.7%]; *P* = .02), prior stroke (31 [2.9%] vs 5 [0.8%]; *P* = .01), gestational diabetes (134 [12.4%] vs 45 [7.4%]; *P* = .002), preeclampsia (40 [3.7%] vs 3 [0.5%]; *P* < .001), and prepartum use of antihypertensive agents (32 [3.0%] vs 5 [0.8%]; *P* = .007) and antiplatelet agents (88 [8.2%] vs 10 [1.7%]; *P* < .001).

**Table 1.** Baseline Characteristics of Women With MMD by Mode of Birth

Characteristic	Cesarean (n = 1077)	Vaginal (n = 606)	Total (N = 1683)	*P* value
Age, mean (SD), y	32.2 (8.0)	36.1 (6.6)	33.6 (7.8)	<.001
Age group, No. (%)				
19-29 y	318 (29.5)	230 (38.0)	548 (32.6)	<.001
30-39 y	708 (65.7)	365 (60.2)	1073 (63.8)	
40-49 y	51 (4.7)	11 (1.8)	62 (3.7)	
Onset type of MMD, No. (%)				
Asymptomatic or nonvascular	685 (63.6)	397 (65.5)	1082 (64.3)	.63
Hemorrhagic onset	130 (12.1)	74 (12.2)	204 (12.1)	
Ischemic onset	262 (24.3)	135 (22.3)	397 (23.6)	
Timing of MMD diagnosis, No. (%)				
Prepregnancy	489 (45.4)	52 (8.6)	541 (32.1)	<.001
During pregnancy	62 (5.8)	10 (1.7)	72 (4.3)	
Puerperium^a^	18 (1.7)	7 (1.2)	25 (1.5)	
Postpuerperium	508 (47.2)	537 (88.6)	1045 (62.1)	
Surgery type				
Direct bypass	82 (7.6)	40 (6.6)	122 (7.2)	.02
Indirect bypass	117 (10.9)	41 (6.8)	158 (9.4)	
Combined bypass^b^	102 (9.5)	71 (11.7)	173 (10.3)	
None	776 (72.1)	454 (74.9)	1230 (73.1)	
Bypass surgery status	301 (27.9)	152 (25.1)	453 (26.9)	.22
Comorbidities^c^				
Diabetes mellitus	16 (1.6)	1 (0.2)	17 (1.1)	.02
Dyslipidemia	8 (0.8)	0	8 (0.5)	.07
Heart failure	34 (3.3)	7 (1.2)	41 (2.6)	.01
Hypertension	25 (2.5)	4 (0.7)	29 (1.8)	.02
Stroke history^d^	31 (2.9)	5 (0.8)	36 (2.1)	.01
Pregnancy-related complications^e^				
Eclampsia	5 (0.5)	1 (0.2)	6 (0.4)	.57
Gestational diabetes	134 (12.4)	45 (7.4)	179 (10.6)	.002
Gestational hypertension	34 (3.2)	12 (2.0)	46 (2.7)	.21
Overt diabetes during pregnancy	8 (0.7)	2 (0.3)	10 (0.6)	.47
Preeclampsia	40 (3.7)	3 (0.5)	43 (2.6)	<.001
Medication use^f^				
Anticoagulants	7 (0.6)	0	7 (0.4)	.11
Antidiabetic agents	18 (1.7)	4 (0.7)	22 (1.3)	.13
Antihypertensive agents	32 (3.0)	5 (0.8)	37 (2.2)	.007
Antiplatelet agents	88 (8.2)	10 (1.7)	98 (5.8)	<.001
Aspirin monotherapy	66 (6.1)	8 (1.3)	74 (4.4)	<.001
Statins	10 (0.9)	1 (0.2)	11 (0.7)	.12

Abbreviation: MMD, moyamoya disease.

^a^ The period up to 6 weeks after giving birth.

^b^ Combined bypass surgery, integrating direct (superficial temporal artery–middle cerebral artery anastomosis) and indirect (encephaloduroarteriosynangiosis or encephalomyosynangiosis) revascularization techniques.

^c^ Comorbidities include diagnoses made within 3 years prior to giving birth.

^d^ Stroke history refers to patients with at least 2 separate diagnostic records of stroke within 2 years before pregnancy, to enhance diagnostic validity.

^e^ Pregnancy-related complications were defined using diagnostic codes recorded during the pregnancy. Multiple pregnancy was also considered, but it was not found during the first pregnancy in any of the patients.

^f^ Medication use refers to patients who were prescribed the corresponding medications for a cumulative duration of 30 days or more within 120 days prior to giving birth.

### Postpartum Stroke Risk According to Mode of Birth

The incidence rates and hazard ratios (HRs) for postpartum stroke were evaluated across multiple follow-up periods (see Table 2 for any stroke and eTable 2 in Supplement 1 for the secondary outcome). For the primary outcome (any stroke), no significant differences between cesarean and vaginal birth were observed in any follow-up periods. Incidence rates (per 1000 person-years [PY]) and adjusted HRs (model 3; cesarean birth as reference) were as follows: 0 to 3 months, 63.49 vs 33.33 per 1000 PY (aHR, 0.71 [95% CI, 0.26-1.97]; *P* = .52); 0 to 1 year, 27.34 vs 16.75 per 1000 PY (aHR, 0.80 [95% CI, 0.35-1.79]; *P* = .58); and 0 to 3 years, 20.37 vs 17.13 per 1000 PY (aHR, 0.90 [95% CI, 0.55-1.47]; *P* = .67). Analyses of ischemic stroke, hemorrhagic stroke, TIA, and hemorrhagic stroke subtypes (subarachnoid and intracerebral hemorrhage) also showed no significant differences (eTable 2 in Supplement 1).

**Table 2.** IRs and HRs for Any Stroke Among Women With Moyamoya Disease by Mode of Birth

Mode of birth	Total No.	No. of Events	No. of PY	IR per 1000 PY	Model 1		Model 2		Model 3	
					Crude HR (95% CI)^a^	*P* value	aHR (95% CI)^b^	*P* value	aHR (95% CI)^c^	*P* value
0 to 3-mo Follow-up										
Cesarean	1077	20	315	63.49	1 [Reference]	.17	1 [Reference]	.18	1 [Reference]	.52
Vaginal	606	6	180	33.33	0.53 (0.21-1.32)		0.53 (0.21-1.33)		0.71 (0.26-1.97)	
1-y Follow-up										
Cesarean	1077	28	1024	27.34	1 [Reference]	.20	1 [Reference]	.22	1 [Reference]	.58
Vaginal	606	10	597	16.75	0.62 (0.30-1.28)		0.64 (0.31-1.32)		0.80 (0.35-1.79)	
3-y Follow-up										
Cesarean	1077	58	2847	20.37	1 [Reference]	.48	1 [Reference]	.61	1 [Reference]	.67
Vaginal	606	30	1751	17.13	0.85 (0.55-1.32)		0.89 (0.57-1.39)		0.90 (0.55-1.47)	

Abbreviations: aHR, adjusted hazard ratio; HR, hazard ratio; IR, incidence rate; PY, person-years.

^a^ Model 1: crude HR (unadjusted).

^b^ Model 2: adjusted for maternal age.

^c^ Model 3: adjusted for maternal age, surgery type, bypass surgery, pregnancy-related complications (eclampsia, gestational diabetes, gestational hypertension, overt diabetes, preeclampsia), prior 2 times stroke within 2 years before pregnancy, and medication use within 120 days before giving birth (anticoagulants, antidiabetic agents, antihypertensive agents, antiplatelet agents including aspirin, and statins).

### Subgroup Analyses of Stroke Risk by Mode of Birth

To assess the consistency of our findings, we performed prespecified subgroup analyses based on maternal age, diagnostic timing of MMD relative to pregnancy, clinical onset type of MMD, bypass surgery status, and type of revascularization surgery during 3-year follow-up, adjusted for model 3 covariates (Table 3). No significant differences between cesarean and vaginal birth were observed across subgroups after Bonferroni correction (corrected *P* > .05). By age group, those aged 19 to 29 years (18.55 vs 10.39 per 1000 PY; aHR, 0.56 [95% CI, 0.23-1.36]; *P* = .20) and those aged 30 to 39 years (21.13 vs 22.03; aHR, 1.06 [95% CI, 0.64-1.78]; *P* = .81) showed no differences; analysis for those aged 40 to 49 years was not feasible (cesarean, 3 of 51 [5.9%]; vaginal, 0 of 11). When stratified by the timing of MMD diagnosis, type of revascularization surgery, and bypass surgery status, there was no significant difference in stroke risk between the modes of birth in any subgroup (eg, postpuerperium: 24.05 vs 17.28 per 1000 PY; aHR, 0.72 [95% CI, 0.44-1.19]; *P* = .20). For the secondary outcomes (ischemic stroke, hemorrhagic stroke [including intracerebral and subarachnoid hemorrhage], and TIA), no significant differences were observed across subgroups after Bonferroni correction (eTable 3 in Supplement 1).

**Table 3.** Subgroup Analyses of HRs for Any Stroke Among Women With MMD by Mode of Birth

Subgroup	aHR (95% CI)^a^	*P* value	No./total No. (%)		Cesarean		Vaginal		*P* value for interaction^b^
			Cesarean	Vaginal	No. of PY	IR per 1000 PY	No. of PY	IR per 1000 PY	
Age group, y									
19-29	0.56 (0.23-1.36)	.20	16/318 (5.0)	7/230 (3.0)	862.5	18.55	673.5	10.39	.22
30-39	1.06 (0.64-1.78)	.81	39/708 (5.5)	23/365 (6.3)	1846.1	21.13	1044.1	22.03	
40-49	NA	NA	3/51 (5.9)	0/11	NA	NA	NA	NA	
Timing of MMD diagnosis									
Prepregnancy	1.24 (0.28-5.45)	.78	14/489 (2.9)	2/52 (3.8)	1195.8	11.71	140.3	14.26	.45
During pregnancy	NA	NA	3/62 (4.8)	0/10	NA	NA	NA	NA	
Puerperium^c^	0.42 (0.05-3.49)	.42	6/18 (33.3)	1/7 (14.3)	35.2	170.45	18.0	55.56	
Postpuerperium	0.72 (0.44-1.19)	.20	35/508 (6.9)	27/537 (5)	1455.2	24.05	1562.3	17.28	
Onset type of MMD									
Asymptomatic or nonvascular onset	0.10 (0.01-0.79)	.03^d^	15/685 (2.2)	1/397 (0.3)	1849.8	8.11	1174.5	0.85	.007^e^
Ischemic onset	1.49 (0.73-3.03)	.27	17/262 (6.5)	14/135 (10.4)	684.6	24.83	380.8	36.76	
Hemorrhagic onset	0.94 (0.50-1.77)	.84	26/130 (20.0)	15/74 (20.3)	313	83.07	195.3	76.80	
Type of revascularization surgery									
No surgery	0.84 (0.49-1.44)	.52	38/776 (4.9)	20/454 (4.4)	2069.1	18.37	1315.8	15.2	.87
Indirect bypass	1.49 (0.36-6.27)	.58	5/117 (4.3)	3/41 (7.3)	284.4	17.58	117.5	25.53	
Direct bypass	0.65 (0.13-3.20)	.59	6/82 (7.3)	2/40 (5.0)	228.3	26.28	114.8	17.42	
Combined bypass^f^	0.75 (0.25-2.23)	.60	9/102 (8.8)	5/71 (7.0)	265.6	33.89	202.5	24.69	
Bypass surgery									
No	0.84 (0.49-1.44)	.52	38/776 (4.9)	20/454 (4.4)	2069.1	18.37	1315.8	15.2	.75
Yes	0.91 (0.42-1.94)	.80	20/301 (6.6)	10/152 (6.6)	778.3	25.7	434.8	23.0	

Abbreviation: aHR, adjusted hazard ratio; HR, hazard ratio; IR, incidence rate; MMD, moyamoya disease; NA, not applicable; PY, person-years.

^a^ Cox proportional hazards regression models for the 3-year postpartum period, adjusted as in model 3 (Table 2).

^b^ The heterogeneity of associations between the mode of birth and outcomes across subgroups.

^c^ The period up to 6 weeks after giving birth.

^d^ *P* = .15 after Bonferroni correction for multiple comparisons.

^e^ *P* = .04 after Bonferroni correction for multiple comparisons.

^f^ Combined bypass surgery, integrating direct (superficial temporal artery–middle cerebral artery anastomosis) and indirect (encephaloduroarteriosynangiosis or encephalomyosynangiosis) revascularization techniques.

However, a significant interaction was observed between the mode of birth and the clinical onset type of MMD for the risk of any stroke (interaction *P* = .04 after Bonferroni correction); the adjusted HR for vaginal vs cesarean birth was 0.10 (95% CI, 0.01-0.79) in the asymptomatic or nonvascular onset subgroup, 1.49 (95% CI, 0.73-3.03) in the ischemic onset subgroup, and 0.94 (95% CI, 0.50-1.77) in the hemorrhagic onset subgroup. In the asymptomatic or nonvascular onset subgroup, the risk of any stroke was nominally lower with the vaginal birth than with the cesarean birth (8.11 vs 0.85 per 1000 PY; aHR, 0.10 [95% CI, 0.01-0.79]; *P* = .03), which was not significant after multiple-testing correction (*P* = .15). No differences were observed in the ischemic onset (24.83 vs 36.76 per 1000 PY; aHR, 1.49 [95% CI, 0.73-3.03]; *P* = .27) or hemorrhagic onset (83.07 vs 76.80 per 1000 PY; aHR, 0.94 [95% CI, 0.50-1.77]; *P* = .84) subgroups.

### Analysis of Time-Varying Associations of Mode of Birth

To assess time-varying associations between the mode of birth and stroke risk, we applied piecewise Cox proportional hazards regression models stratified by postpartum intervals (≤180 days, 181-365 days, 366-730 days, and >730 days) (Table 4) adjusted for covariates as in model 3 (with cesarean birth as reference). We first examined the overall temporal pattern of stroke incidence following childbirth. The incidence rate for any stroke was highest within the 180 days postpartum (35.7 per 1000 PY) and decreased thereafter to 11.1 per 1000 PY at 181 to 365 days, 16.3 per 1000 PY at 366 to 730 days, and 17.3 per 1000 PY beyond 730 days. For any stroke, no significant differences between the modes of birth were observed in any interval: 180 days or less (44.62 vs 20.26; aHR, 0.49 [95% CI, 0.19-1.23]; *P* = .13); 181 to 365 days (9.84 vs 13.30; aHR, 1.41 [95% CI, 0.37-5.33]; *P* = .62); 366 to 730 days (14.74 vs 18.87; aHR, 1.36 [95% CI, 0.60-3.08]; *P* = .46); and more than 730 days (18.34 vs 15.80; aHR, 0.90 [95% CI, 0.39-2.08]; *P* = .80). The highest incidence rates occurred in the early postpartum period (≤180 days), with a trend toward stabilization thereafter, but the mode of birth did not modify this temporal pattern. Results for ischemic stroke, hemorrhagic stroke (including subarachnoid and intracerebral hemorrhage), and TIA were consistent (eTable 4 in Supplement 1).

**Table 4.** Piecewise HRs for Any Stroke Post Partum Among Women With Moyamoya Disease by Time Interval

Interval, d	Total No.	No. of Events	No. of PY	IR per 1000 PY	aHR (95% CI)^a^	*P* value
≤180						
Overall	1683	29	811.6	35.7	0.49 (0.19-1.23)	.13
Cesarean	1077	23	515.5	44.62	NA	NA
Vaginal	606	6	296.1	20.26	NA	NA
181-365						
Overall	1623	9	809	11.1	1.41 (0.37-5.33)	.62
Cesarean	1024	5	508.3	9.84	NA	NA
Vaginal	599	4	300.8	13.3	NA	NA
366-730						
Overall	1576	25	1532.4	16.3	1.36 (0.60-3.08)	.46
Cesarean	984	14	949.5	14.74	NA	NA
Vaginal	592	11	582.9	18.87	NA	NA
>730						
Overall	1487	25	1441.8	17.3	0.90 (0.39-2.08)	.80
Cesarean	910	16	872.2	18.34	NA	NA
Vaginal	577	9	569.6	15.8	NA	NA

Abbreviations: aHR, adjusted hazard ratio; HR, hazard ratio; IR, incidence rate; PY, person-years.

^a^ Piecewise Cox proportional hazards regression models for the 3-year postpartum period, adjusted as in model 3 (Table 2).

## Discussion

This nationwide cohort study found that stroke risk after childbirth among women with MMD does not differ significantly between cesarean and vaginal births across time intervals and subgroups. However, risk peaked in the early postpartum period, underscoring the need for vigilant monitoring and preventive strategies during this vulnerable window. Importantly, a significant interaction was observed between the mode of birth and MMD onset type. Overall, vaginal birth is generally safe, but tailored decisions based on onset characteristics are essential to optimize maternal outcomes in MMD.

Several previous studies have investigated and compared the safety of cesarean vs vaginal birth among pregnant women with MMD.^10,11,12,13,15,16,18,28^ Most of these studies are limited by small sample sizes, single-center designs, or reliance on case reports and case series. The largest study to date, by Takahashi et al^18^ in Japan, used a survey-based approach to analyze peripartum stroke occurrence over the preceding 5 years. That study included data from 2 nationwide surveys: a facility-based survey (64 births across 270 perinatal medical centers) and a patient-based survey (278 births from 148 patients). Antoniazzi et al^28^ analyzed 97 births from a US inpatient database and found no significant difference in arterial ischemic stroke between the modes of birth. Other studies have been small-scale, single-center investigations.^6,7,8,9,11,12^ Our nationwide cohort of pregnant women with MMD, with comprehensive 3-year follow-up provides the first, to our knowledge, robust, population-level evidence of no overall association between the mode of birth and stroke risk, which may offer clinical guidance for birth planning in this high-risk population.

We found no significant difference in the risk of stroke or TIA between cesarean and vaginal births across time intervals and subgroups. Cesarean birth has traditionally been preferred in this vulnerable population because of concerns about hemodynamic stress.^17,18,19^ Although it offers controlled conditions,^30^ a cesarean birth increases long-term risks such as pelvic adhesion, chronic pelvic pain, placenta previa, placenta accreta, and uterine rupture compared with a vaginal birth.^31,32,33,34^ Meanwhile, a vaginal birth has the advantages of lower maternal morbidity and faster recovery.^31,32,35^ Our findings support that, with appropriate management (eg, epidural anesthesia), vaginal birth is not associated with a higher risk of adverse cerebrovascular outcomes.^11,12,13^ Birth planning for women with MMD should be individualized, rather than defaulting to cesarean birth.

In our subgroup analysis, a statistically significant interaction was observed between the mode of birth and the onset type of MMD for postpartum stroke risk within 3 years. Specifically, the HRs varied substantially by onset type; vaginal birth was associated with a markedly lower risk in the asymptomatic or nonvascular group (aHR, 0.10), whereas no such benefit—and potentially an opposing trend—was observed in the ischemic onset group (aHR, 1.49) or hemorrhagic onset group (aHR, 0.94), highlighting the potential heterogeneity of postpartum cerebrovascular outcomes and underscoring the need for further investigation to replicate these results and clarify underlying mechanisms. Indeed, the trend toward a lower risk of stroke with vaginal birth for patients with asymptomatic or nonvascular onset MMD supports the importance of considering onset type as a key factor when determining birth strategies. These results are consistent with previous studies suggesting that vaginal birth may be reasonable for patients with favorable cerebral perfusion status detected on single-photon emission computed tomography scan or for those without TIA or stroke in the preceding year.^14^ Therefore, incorporating clinical markers such as onset type, cerebral perfusion status, and symptom-free duration may provide an important foundation for developing individualized birth strategies for women with MMD.

The findings of this study provide evidence that, in the absence of obstetric indications for cesarean birth, vaginal birth is considered a reasonable option for pregnant women with MMD. However, further research is needed to improve pregnancy and childbirth outcomes in this population. First, studies should refine the indications for mode of birth by identifying patient-specific factors—such as cerebral hemodynamic status and vascular reserve—that influence whether vaginal birth is relatively safe or cesarean birth is preferable.^14^ Second, clinical research is needed to develop strategies to minimize stroke risk at different stages—during pregnancy, childbirth, and the postpartum period—especially for patients with MMD before conception. In our study, the incidence rate of any stroke was 35.7 per 1000 PY within 180 days after giving birth, decreasing to 11.1 at 181 to 365 days, 16.3 at 366 to 730 days, and 17.3 at more than 730 days (Table 4). These findings suggest that mothers with MMD are particularly vulnerable to stroke during the peripartum period, underscoring the need for tailored management strategies. Such strategies may include the use of antiplatelet agents, optimized blood pressure and hemodynamic control, and assisted birth techniques.^12,14,36,37,38,39^ Finally, studies incorporating long-term neurological outcomes could help establish guidelines and improve prognosis for pregnant women with MMD.

### Limitations

This study has several limitations. Most importantly, a key limitation of this study is that many women in the cesarean group received a diagnosis of MMD before or during pregnancy, whereas most in the vaginal group received a diagnosis postpuerperium, raising potential selection bias. However, nationwide data minimized selection bias, and subgroup analyses according to the timing of MMD diagnosis (prepregnancy, during pregnancy, puerperium, and postpuerperium) also showed comparable results, although the sample size of patients with MMD in the vaginal birth group is relatively small, resulting in wide 95% CIs. Second, baseline differences existed between groups (eg, age, risk factors, preeclampsia, and medication), but adjusted analyses remained robust. Third, the retrospective design may have introduced selection bias and unmeasured confounding. Fourth, claims data lacked detailed clinical, radiological, and genetic information (eg, angiographic severity; unilateral or bilateral involvement and specific vessel involvement [internal carotid artery or middle cerebral artery]; perfusion status; *RNF213* status; and intrapartum management), which could affect stroke risk. Fifth, detailed peripartum clinical information was not fully available in the claims database. Specifically, we could not distinguish cases where an attempted vaginal birth was converted to an emergency cesarean birth, nor could we access data on anesthetic management (eg, general vs spinal anesthesia) or precise intrapartum blood pressure control. Sixth, the choice of birth mode for patients with MMD is complex and influenced by various factors, including individual clinical judgment, institutional protocols, and history of stroke or other comorbidities. Although we adjusted for several measured confounders using multivariable models, our study is subject to potential selection bias and unmeasured confounding, as these qualitative factors are not fully captured in a claims-based database. Specifically, patients perceived to be at higher risk by clinicians may have been more likely to undergo planned cesarean birth, which could influence the observed outcomes. Finally, this study focused on major clinical events such as stroke and mortality; however, information on milder neurological events, functional status, and long-term maternal and fetal outcomes was not available in the claims database. Consequently, the true risk profile associated with each mode of birth—particularly regarding nondisabling symptoms or quality-of-life measures—might have been underestimated.

## Conclusions

In this cohort study of women with MMD, the mode of birth was not significantly associated with the risk of postpartum stroke, although the association varied according to the clinical onset type of MMD. The incidence of stroke peaked during the early postpartum period, regardless of the mode of birth. These findings suggest that vaginal birth could be considered a reasonable option for women with MMD in the absence of specific obstetric indications for cesarean birth. Decisions regarding the mode of birth should be individualized, particularly according to the clinical onset type. Last, vigilant monitoring during the early postpartum period remains warranted to minimize stroke risk in this high-risk population.

## Notes

Supplement 1. **eTable 1.** Definition of Variables and Corresponding ICD-10/Procedure Codes **eTable 2.** Incidence Rates and Hazard Ratios by Mode of Birth in Women With Moyamoya Disease **eTable 3.** Subgroup Analyses of Hazard Ratios by Mode of Birth in Women with Moyamoya Disease **eTable 4.** Piecewise Hazard Ratios by Time Intervals in Women With Moyamoya Disease

Supplement 2.
Data Sharing Statement
