# Supplementary material for: Mode of Birth and Stroke Risk After Childbirth Among Women With Moyamoya Disease
Source: JAMA Netw Open. 2026 Mar 23;9(3):e263112. doi: 10.1001/jamanetworkopen.2026.3112 (PMC13010194; doi:10.1001/jamanetworkopen.2026.3112)
Supplement: Supplement 2. — Data Sharing Statement [file jamanetwopen-e263112-s002.pdf]

Data Sharing Statement

Kim. Mode of Birth and Stroke Risk After Childbirth Among Women With Moyamoya Disease. *JAMA Netw Open*. Published March 23, 2026. doi:10.1001/jamanetworkopen.2026.3112

Data
Data available: No

Additional Information
Explanation for why data not available: This is a population-based study using National Health Claim Data. There is an authorization process and a barrier to assess the data.
